# Femicide in Turkey between 2000 and 2010

**DOI:** 10.1371/journal.pone.0182409

**Published:** 2017-08-23

**Authors:** Sadik Toprak, Gokhan Ersoy

**Affiliations:** 1 Department of Forensic Medicine, Bulent Ecevit University, Zonguldak, Turkey; 2 Institute of Forensic Medicine, Istanbul University, Istanbul, Turkey; PhD, London School of Hygiene & Tropical Medicine, UNITED KINGDOM

## Abstract

Although intimate partner violence (IPV) is an important problem that threatens women’s health, very few studies focus on the victim—perpetrator relationship or examine this relationship across Turkey. The aim of this study is to contribute to a better understanding of femicide cases in Turkey and to describe the socio-demographic, clinical, forensic, and criminological characteristics of femicide victims and offenders. This study analysed 162 femicide cases that occurred in 12 cities in Turkey from 1 January 2000 to 31 December 2010. Eighty women were killed by their partners (classified as intimate partner femicide, IPF), and 81 women were killed by one of their relatives, friends, or strangers (classified as non-intimate partner femicide, non-IPF). According to our results, the typical IPF victim is of child-bearing age, does not have a paid job, is married or divorced, is killed in a domestic setting due to injuries to the thorax or abdomen produced by an edged/pointed weapon or firearm, and is possibly a victim of overkill. The typical IPF perpetrator is close to his victim’s age, has a paid job, has no mental disability, owns a gun, and has threatened his partner or ex-partner previously because of jealousy/infidelity/honour or separation. The typical non-IPF victim is very similar to the IPF victim; however, her marital status can be single, married or divorced, and she is commonly killed by a relative. The surveillance and screening of femicide and IPV is an important step when analysing and attempting to prevent femicide. Second, the training and sensitization of health professionals are important. Moreover, health staff should be encouraged to participate in advocacy interventions. Third, gun ownership must be brought under control.

## Introduction

Violence against women is a violation of human dignity, and, in its worst form, femicide violates the right to life [1]. The World Health Organization (WHO) defines femicide as follows: “Femicide is generally understood to involve the intentional murder of women because they are women, but broader definitions include any killings of women or girls” [2]. Globally, 13.5% of homicide cases are committed by an intimate partner. However, the proportion of femicide cases is six times higher than the proportion of men killed by their partners (38.6% and 6.3% of female and male homicides, respectively) [3].

The WHO addresses 4 different types of femicide: intimate femicide (committed by a current or former husband or boyfriend); non-intimate femicide; “honour killing” (when the perpetrator claims that his motive is to preserve/restore his family’s honour, that is, for reasons related to the victim’s known or suspected culturally defined sexual impropriety); and dowry-related femicide (the husband and in-laws kill the young bride when dowry demands are not met) [2,4,5]. In fact, the terms “intimate” and “non-intimate” refer to the relationship between the victim and perpetrator(s), whereas the other terms suggest cultural or individual motives. In a systematic review, Stöckl et al. showed that 43% of female homicides were committed by intimate partners, and this percentage was higher in South Asia and lower in Eastern Mediterranean (62% and 20%, respectively) [3]. The percentage in Turkey was reported to be 78.2%; however, this figure is of limited value because the search methodology was based on news reports [6].

Separation and estrangement are major motives for intimate homicides [7,8,9]. Further, the number of estrangement-related homicides may be underestimated, as many separated couples may be perceived as intact because of the long, drawn-out process of separating [8]. By contrast, honour-based femicide can be understood as both intimate and non-intimate femicide depending on the killer—victim relationship, especially when family members participate in this crime [10]. Dowry-related homicide has shown unique regional patterns, which are based on cultural factors. This trend is observed in particular Indian regions, where arguments and dowry are reported as major risk factors for femicide [5]. More studies comparing the relationships involved in and motives for femicide should be conducted. Femicide is considered a public health issue and a legal matter, and more data are needed to establish preventative measures [11]. However, despite the magnitude of this problem, research has mainly focused on non-fatal intimate partner violence (IPF) rather than femicide [12]. Moreover, data on the victim—offender relationship, even in countries with advanced homicide monitoring data systems, are scarce [3]. In developing countries such as Turkey, the data on femicide is limited [13], and very few studies focus on the victim—offender relationship [1,14,15].

A number of intermediate factors that cut across all typologies may also play a role in how femicides are perpetrated. These factors include the psychoactive substances and weapons involved in these crimes. A better understanding of the influence of the femicide mechanism in facilitating femicide can be of great value to the development of femicide prevention policies, which, when appropriately targeting such elements, can reduce violence before it becomes lethal. A study conducted in Portugal found that weapons, including firearms and sharp objects, were used in 72.6% of femicide cases [12]. Another study conducted in Italy showed that weapons were used in 79.4% of femicide cases [16]. According to studies conducted in Turkey, weapon use in femicide cases ranged between 66.5%, 80%, 80.1% and 83.4% [14,15,17,18].

The aim of the study was to contribute to a better understanding of femicide cases in Turkey and to describe the socio-demographic, clinical, forensic and criminological characteristics of femicide victims and perpetrators.

## Materials and methods

In this retrospective study, data were collected from three sources: court files, police records and forensic autopsy records. This study analysed 162 femicides that occurred in 12 cities of Turkey from 1 January 2000 to 31 January 2010.

The data were collected according to the Nomenclature of Territorial Units for Statistics (NUTS), created by the European Statistics Office (EUROSTAT) and accepted by the European Union [19]. NUTS was created to break down the economic territory of the European Union into territorial units to generate regional statistics and to target political interventions at the regional level [20]. NUTS has three layers, and NUTS-1 (major socio-economic regions) was used in this study. In this study, the selected territorial units were Istanbul, West Marmara, Aegean, East Marmara, West Anatolia, the Mediterranean, Central Anatolia, the West Black Sea, the East Black Sea, Northeast Anatolia, Central East Anatolia and Southeast Anatolia.

### Sample size determination and sampling technique

A one-stage cluster sampling method was used to select the sample. The primary units of sampling were 12 territorial units. Turkey has 305 criminal courts [21], and each territorial unit has between 21 and 32 criminal courts. A random, computer-generated number was used to select one criminal court from each territorial unit. From each of the selected criminal courts, all cases between 1 January 2000 and 31 December 2010 were included in the study. The cases selected were concluded by 31 December 2010 to ensure that the criminal investigation and judicial decisions had been confirmed.

According to the United Nations Office on Drugs and Crime, there were 3400 intentional female homicide victims in Turkey between 2003 and 2010 (the annual average is 425) [22,23]. The expected number of femicide cases was calculated to be 4675 for the 2000 to 2010 period.

Previous studies have shown that the percentage of women killed with any weapon (including those with firearm and sharp force injuries) was 83.4% [19].

The sample size was estimated using Epi Info 2000 Statcalc software with a known prevalence of 83.4% and a 78% worst acceptable prevalence within the 95% CI from a total population of 4675 (Centers for Disease Control, Atlanta, GA). Accordingly, the minimum sample size (n) was found to be 172. One hundred eighty-two cases were collected from 12 criminal courts; however, 20 cases were excluded due to missing information about the autopsy, resulting in a final sample size of 162.

The ethics committee of Bulent Ecevit University approved the study protocol (ethics code 2014-151-02/09). All data were analysed anonymously; therefore, informed consent for each individual was neither necessary nor possible. This research was supported by the Scientific and Technological Research Council of Turkey (TUBITAK) (Project no: 115S024).

### Statistical analysis

The chi-square test and Fisher’s exact test were used for categorical variables, and the t-test was used for non-categorical variables. The accepted significance level was p<0.05. All statistical analyses were performed using the Epi Info 2000 (Centers for Disease Control, Atlanta, GA).

## Results

### Characteristics of the perpetrators

A total of 162 events resulting in 161 femicides in 12 cities were analysed as a representative sample of femicide in Turkey. There were 155 perpetrators and 161 victims; one victim was killed by two perpetrators, and in seven cases, the perpetrator killed two women.

In non-IPF cases, 9 of 76 (11.8%) perpetrators were female (three close relatives, two daughters, and three more distant relatives or friends). Of the IPF perpetrators, 75% (n = 57) were between 21 and 50 years of age. However, the non-IPF perpetrators were approximately 10 years younger; 79% of the perpetrators were between 15 and 40 years of age. The mean age of the perpetrators was between 32.3±13.4 and 39.6±13.7 years for the non-IPF and IPF groups, and there was a statistical significance (p = 0.001) (Table 1).

**Table 1:** Socio-demographic characteristics of perpetrators and general risk factors for homicide, by group. ld: not analysed due to limited data; na: not analysed; ns: no significance.

	Total samples	Non-IPF (n = 76)	IPF (n = 79)	p
Mean age	36.5 ±17.8	39.6±13.7	32.3±13.4	<.001
Gender (Female/Male)	9/155	9/67 (11.8%)	0/79(0)	
Range^a^		16–77	17–80	
	%^b^	n (%)^b^	n (%)^b^	
0–7	-	-	-	na
15–20	12.2	15(19.7)	4(5.1)	
21–30	27.7	24(31.5)	19(24.1)	
31–40	24.5	18(23.7)	20(25.3)	
41–50	15.4	6(7.9)	18(22.8)	
51+	14.9	9(11.8)	15(19)	
High level of education^c^				
Yes	5.8	2(2.6)	7(8.9)	ns
No	49	43(56.6)	33(41.8)	
Marital status				
Single	23.9	23(30.2)	14(17.7)	<.05
Married	58.7	37(48.7)	54(68.3)	
Divorced	5.1	2(2.6)	6(7.6)	
Employment				
Employed	46.4	30(39.5)	42(53.2)	<.05
Retired—Student^d^	12.9	8(10.5)	12(15.2)	
Unemployment	20	21(27.6)	10(12.6)	
Mental disorder				
Yes	8.4	5(6.6)	8(10.1)	ns
No	91.6	71(93.4)	71(89.9)	
Substance use for known cases				
Alcohol	2.6	1(1.3)	3(3.8)	ld
Cannabis	2.6	-	4(5.1)	
No substance	21.9	13(17.1)	21(26.6)	
Previous criminal records				
Yes	32.3	23(30.3)	27(34.2)	ns
No	42.6	27(35.5)	39(49.4)	

^a^: No case involved a perpetrator between the ages of 8 and 14.

^b^: Percentages may not add up to 100% due to the missing information.

^c^: High school diploma and above.

^d^: Four students were perpetrators.

### Characteristics of the victims

The mean ages of the IPF victims and non-IPF victims were 34.1±11.7 and 39.01±22.1 years of age, respectively. No victim was between 8 and 14 years old. Although there was no significant intergroup difference in the mean age, victims over 51 years of age significantly increased in the non-IPF group, which also included nine minors younger than 10 years of age. If these minors were excluded, the mean age of the non-IPF group increased to 44.0±19.5. By contrast, all but five IPF victims were between 15 and 50 years of age. (Table 2) Similarly, if perpetrators were close relatives, the proportion of victims under 51 years old was 83.3%.

**Table 2:** Socio-demographic characteristics of victims and general risk factors for homicide, by group. ld: not analysed due to limited data; na: not analysed; ns: no significance.

	Total Samples	Non IPF (n = 81)	IPF (n = 80)	p
Mean age		39.0±22.1	34.1±11.7	ns
Range^a^		0–83	15–72	ns
	%^b^	n(%)^b^	n(%)^b^	
0–7	5.6	9(11.1)	-	
15–20	10.6	6(7.4)	11(13.8)	
21–30	23.6	17(21)	21(26.2)	
31–40	22.4	16(19.8)	20(25)	
41–50	18.6	7(8.6)	23(28.8)	
51+	19.2	26(32.1)	5(6.2)	
High level of education^c^				
Yes	3.7	3(3.7)	3(3.7)	ld
No	23.4	13(16)	6(7.5)	
Marital Status				
Single	19.9	18(22.2)	14(17.5)	<.001
Married	47.2	25(30.7)	54(67.5)	
Divorced	18.6	21(25.9)	9 (11.2)	
Employment				
Employed	12.4	9(11.1)	11(13.8)	<.05
Retired—Student^d^	2.4	3(3.7)	1(1.2)	
Housewife	29.2	22(27.2)	25(31.2)	
Unemployment	11.2	9(11.1)	9(11.2)	
Link with perpetrators				
Spouses	29.8	-	48(60)	na
Ex-spouse	1.2		2(2.5)	
Date	13.7		22(27.5)	
Ex-Date	3.7		6(7.5)	
Religious marriage	1.2		2(2.5)	
Stranger^e^	13	21(25.6)	-	
Close Relatives/Siblings	19.9	32(39)		
Offsprings	3.1	5(6.1)		
Distant Relatives	4.3	7(6.5)		
Friends/Neighbour	7.4	12(14.6)		
Unnknown	3.1	5(6.1)		
Previous abuse history				
Physical abuse	16.1	5(6.1)	21(26.2)	ld
Sexual abuse	4.3	4(4.9)	3(3.7)	
Emotional abuse	Unknown			
Mental Disorder				
Yes	0.6	-	1(1.2)	ld
No	99.4	81(100)	79(98.8)	
Substance use for cases				
Alcohol	1.2	-	2(1.5)	na
Codein	0.6	1(1.2)	-	
Chloroform	1.2	-	2(1.5)	
No substance	97	80(98.8)	76(97)	

^a^: There were no case between the ages of 8–14 years.

^b^: Percentages may not add up to 100% due to missing information.

^c^: Post-secondary studies and/or degree(s)

^d^: Student counts for victims were 2.

^e^: One of the non IPF victim was killed by two strangers.

The mean age differed depending on the motive for the killing. The mean ages of victims were 50.2 ± 4.9 and 38.9±2.8 if the motives were theft and domestic altercations, respectively, whereas they were 30.9±2.5 and 33.2±1.6 if the motives related to separation and jealousy/infidelity, respectively. Theft and domestic altercations constituted 85.7% (n = 24) of the known victims over 50 years of age.

Although 16% of the IPV victims and 28% of the non-IPV victims had no children, no significant intergroup difference was found with regard to having children. At their time of the death, only 10 women in each group (12%) were working actively, but most women (87.5%) were not employed in paid job (i.e., they were housewives or unemployed).

According to previous medical records, only one non-IPF victim was diagnosed with an adjustment disorder. A moderate level of alcohol was detected in two IPF victims. Codeine was found in one IPF victim; however, the amount fell within the therapeutic limits. Chloroform was detected in two non-IPF victims. These two victims had lost consciousness due to chloroform inhalation, and they were killed by manual strangulation.

### Characteristics of the femicide

The age difference showed an important pattern in our dataset: while perpetrators were 5.3±9.8 years older than their victims in IPF cases, perpetrators were 6.6±26.4 years younger than their victims in non-IPF cases.

Typically, the victims of femicide knew the perpetrator(s), except in cases motivated by theft. Spouses (30.6%) were the leading group of perpetrators, followed by relatives/siblings/offspring and boyfriends. The proportion of unknown perpetrators was 13.4% in all cases, but that number increased to 27.2% in non-IPF cases. Detailed documentation is given in Table 1.

At the time of the homicide, 48.6% of perpetrators were living in the same house as their victims (for the cases in which such information is known) (n = 71). In addition, 65.7% of close relatives/siblings/offspring and 62.5% of partners (n = 50) were sharing the same house as the victim. This ratio was 81.2% for spouses and 40.8% for boyfriends (39 and 9 cases, respectively). Table 3 provides detailed information about the victim—perpetrator links. The mean duration of cohabitation with partners was 11.56±11.7 years (range 0.1–40 years). The average separation period was 96.7±154.1 days (range 1–720 days). Ninety per cent of women who were killed by ex-partners were murdered within four months of their separation.

**Table 3:** Ratio of living together with victim among perpetrators.

		Same home	Different home	Unknown	TOTAL	Cumulative Percent in Groups
IPF	Spouses	9	39	-	48	90%
	Dates	13	9	-	22	
	Religious marriage	0	2	-	2	
	Ex-Date	6	0	-	6	10%
	Ex-spouse	2	0	-	2	
	TOTAL					100%
Non IPF	Offsprings	0	3	2	5	6.5%
	Close relatives-siblings	12	20	-	32	50.7%
	Distant relatives	7	0	-	7	
	Neighbours	7	2	-	9	15.6%
	Friends	-	-	3	3	
	Strangers	15	0	6	21	27.2%
	TOTAL					100%

In 21 cases (26.2%), IPF perpetrators had threatened to kill their victims previously and/or immediately before the homicide. Non-IPF perpetrators made fewer threats either previously or at the time of the murder (3.7% and 7.3%, respectively) (Table 3). One woman was killed after taking out a protection order against her husband. In four cases, previous protection orders had expired.

Most of the femicides were committed in a domestic setting; 120 of all cases (74.5%) and 59 of the IPF cases (73.7%) occurred in the victim’s home. Other common settings were on the street (13 cases– 16.2%) and in the countryside/woodlands (four cases– 5%). Two murders were committed on the street (2.5%), and another two were committed in a car (2.5%). Non-IPF cases revealed similar patterns in terms of the scene of the crime.

### Cause of death

Edged and pointed weapons and firearm injuries were the most common causes of death (Table 4). Firearms were used to kill in 75% of the femicides. In terms of the type of firearm, handguns were responsible for a large proportion of deaths in the IPF group compared with other groups. Although non-IPV murders showed the same predominance of firearm use, death due to blunt trauma and strangulation (27.2%) was higher in this group than in other groups at a clear level of statistical significance (p<0.05). Assaults with firearms and edged and pointed weapons also resulted in high fatality rates: 60 of the 68 firearm assaults and 61 of 66 edged and pointed weapons assaults resulted in death due to these injuries.

**Table 4:** Case distribution by cause of death.

Cause of Death	Number of cases N = 161		
	Non IPV	IPV	Total
Edged and pointed weapons	31	30	61
Firearms Handgun Shotgun	11 12	28 9	39 21
Strangulation Manual Ligature	6 6	7 1	13 7
Blunt object	10	4	14
Drowning	1	-	1
Unknown	4	1	5
Total	81	80	161

### Type of wounds

Edged and pointed weapons and firearm injuries were also the most common type of injuries, and ligature marks were the least common (S1 Table). Edged and pointed weapons resulted in the most injuries, with 4.7 and 3.9 injuries per person for IPV and non-IPV, respectively. Blunt injuries were found in 28 cases, whereas death was attributed to blunt injury in only 14 of these cases. Although most cases involved only one type of assault, two or more types of assault occurred in 17 cases, and 16 of these 17 cases had a combination of blunt trauma and another type of assault. Knife wounds and blunt trauma constituted the most common combination (10 cases).

### Injury locations

The head was the most common target for firearm wounds: 56 wounds were found on 42 victims. By contrast, the thoracic region was the most common target for edged and pointed weapon wounds: 247 wounds were found on 47 victims (S1 Table). In general, the thorax and head were the most common injury locations (29.9% vs. 19.3%, respectively). There is statistical significance with regard to region—wound type relationship. The amount of blunt trauma and gunshots in the head region was high, whereas the edged and pointed weapon injuries were usually located in other regions (p<0.00; ligature marks were excluded from the analysis due to their scarcity).

Nineteen (25%) partner homicides were due to *separation*, although the most common motives for known IPF cases were *jealousy/infidelity/honour* (36 cases– 47.4%). However, the motives for the non-IPF victims were different, the most common ones being *domestic altercations* (27 cases– 42.2%) and the *robbery/sexual cases*, which included criminal activity, financial issues or sexual assault cases (22 cases, 32.8%; six of the cases involved sexual assault). There were no reported cases of sexual assault in the IPV group (Table 5). Table 5 was adapted from Block et al. [24].

**Table 5:** Motives and mean ages of victims and perpetrators.

	Non IPF	IPF	Mean ages		P (for ages)^a^
	n (%)	n (%)			
Seperation	-	19 (23.8)	30.9±2.5	36.8±3.3	<.05
Jealousy. infidelity and honour killings	18 (22)	36 (45)	33.2±1.6	39.1±2.0	
General domestic altercations	27 (32.9)	17 (21.3)	38.9±2.8	35.0±2.0	
Theft and other criminal reasons (criminal activities. financial issues or sexual assault)	22 (26.8)	2 (2.5)	50.2±4.9	28.3±1.9	
Mental disease related	2 (2.4)	2 (2.5)		41.2±11.2	
Unknown or unclassified	13 (15.9)	4 (5)			
TOTAL	82	80			

^a^:mental disease related cases were excluded from analysis due to scarcity.

We also analysed how motives related to assault types and target body regions. In the case of assault types, the firearm and edged and pointed weapon injuries were the leading types of wounds in deaths related to separation (17 cases; 94.5%), jealousy/infidelity (41 cases; 80.4%) and domestic altercations (38 cases; 90.5%), whereas few firearm deaths occurred in the “other motives” group. The ratio of gun use to other weapon use in the assaults showed statistically significant intergroup differences based on the cause of death. Assaults with guns (firearm or edged and pointed weapons) were common in all motive groups, except for the other crime group (theft and sexual abuse), for whom assault with bodily force (blunt trauma, strangulation) was more common (p = 0.001) (Table 6).

**Table 6:** Distribution of assault types between the motive types.

	Assault without gun	Assault with gun
	n (%)	n (%)
Separation	2 (7.4)	17 (15)
Jealousy. infidelity and honour killings	8 (29.6)	43 (38.1)
General domestic altercations	5 (18.5)	38 (33.6)
Other reasons (criminal activities. financial strains or sexual assault)	12 (44.4)	15 (13.3)

“Others” consist of robberies in addition to the 6 cases of sexual assaults (of which half (n = 3) were comitted by gun threat).

There was no relationship between the motive type and the injury location, the type of injury and the number of injuries, except that neck injuries were high in the *theft* and *sexual assault motive* groups (69.5%), where lesions due to ecchymosis caused by strangulation were common. By contrast, in cases of blunt trauma in *general domestic altercations* (n = 8), there were less than two examples of ecchymosis.

In our study, there were four filicides among the non-IPF cases. Two mothers killed their new-born babies by starving them. The motive in the first case was the illegitimacy of the child, and the motive in the second case was unknown. Additionally, two mothers were killed with sharp objects at the hands of their four- and six-year-old children. There were two matricide cases in our sample. The first case was an honour killing. In the second case, a young male perpetrator killed both his mother and his sister by manual strangulation after drugging them with chloroform.

## Discussion

Our results showed that approximately half of the femicide cases in Turkey involved intimate partners. Similar results have been found locally and globally in recent studies [3]. This study is the first autopsy-based and judicially proven nationwide study of IPF victims and perpetrators. The purpose of this study was to determine the main characteristics of femicide across Turkey.

### Perpetrators

Half of the homicides with female victims fell into in the IPF category, and the women were killed by their partners at the time of their deaths. Spouses were the leading perpetrators of femicide. Spouses’ rates of femicide were more than the twice that of strangers. If the perpetrator was not a partner, he was a close relative/sibling or offspring in 60% of cases. As observed here, strangers were not in the leading group, even in non-IPF cases, as they trailed close relatives/siblings. This finding is completely consistent with previous findings showing that most homicide victims are killed by a loved one [25]. For example, Sorenson’s analysis of the Supplementary Homicide Reports of the Federal Bureau of Investigation (U.S. Department of Justice) revealed that the average number of femicides committed by strangers was even lower than the average number of IPF cases committed with a handgun [26]. Moreover, if distant relatives and friends (approximately 11% of all cases) were included, most perpetrators were known to the victim.

Previous studies from Turkey revealed that most victims and perpetrators (between 75.9% and 80%) had ongoing or dissolved marriages or relationships [15,17]. We found very similar results, as 81% of IPF victims had been or were married at the time of their deaths. In a local study in Turkey, 52% of femicide victims were reported to be married; however, this study evaluated all kinds of femicide cases [14]. In connection with this finding, IPF perpetrators were mostly married (or divorced), and, as expected, the ratio of single perpetrators was higher in non-IPF cases.

At first glance, it is unclear whether our study supports the generally accepted knowledge that estrangement is a factor that increase the risk of femicide [7]. Although 81.2% of the murdering spouses in our study were cohabitating with their victims, the ratio of cohabitating estranged spouses was 4.31, which is almost equal to the annual marriage-to-divorce ratio [27]. Therefore, additional studies are needed to reveal whether estrangement presents greater risks of IPF in Turkish society.

Most partners lived with the victim; moreover, the percentage for close relatives who lived in the same house as the victim was close to that of partners (more than 60%). Similar results were reported in a recent study [12]. In line with all these results and as expected, the most common crime scene was a domestic setting (74.5%), and the same has been found to apply in several national and international studies [12,14,17]. In our study, the second most common crime scene was on the streets (16.2%), and the same pattern has been found in other Turkish studies [17,18].

The peak age for femicide victims has been found to be 30–49 in some international and local studies [14,15,17,18,28]. Similarly, the median age of victims was placed in the fourth decade in our study. The victim was older than 50 and killed by a partner or close relative(s) in very few cases of femicide (6.3% and 16.7%, respectively).

Although the mean ages of perpetrators in the fourth decade (like victims) and similar results were found in other studies [14], non-IPF perpetrators were significantly younger than IPF perpetrators. Our results showed that both IPF and non-IPF perpetrators were older than those in Yılmaz’s study, which was performed in Turkey at the same time as some international studies [17]. For example, Breitman claimed that the intimate partner homicide risk increased especially among two groups: men who were 16 years older than women or women who were 10 years older than men, irrespective of the offender’s sex [29]. Another study asserted that age discrepancies and the rate of uxoricide increased in parallel [30]. In our study, IPF perpetrators were approximately 5.3 years older than their victims, whereas they were 10 years younger in non-IPF cases. Pereira et al. spotted almost the same trend in their study [12]. This result implies a higher risk of IPF among couples with age discrepancies, which supposedly derives from the dissolution of the relationship [29,30].

The typical perpetrator of femicide is a person with a low level of education, but he is usually employed, although the unemployment rate is higher in the non-IPF group. One of the strongest socio-demographic risk factors for IPF is the perpetrator’s lack of employment (44.8%), as shown in a US study [9]. However, in our study, the unemployment rate was much lower among IPF perpetrators (12.5%) compared with non-IPF perpetrators (22%). The national unemployment rate in Turkey reached 10.1% in 2015 [31]. Therefore, the unemployment rate among IPF perpetrators was slightly higher than the national average. Similarly, the unemployment rate among the perpetrators was found to be between 20 and 26% in studies based in Turkey [14,17].

The prevalence of mental disorders was apparently low in the IPF and non-IPF groups. Although our data showed that 14 (8.6%) perpetrators had been diagnosed with some sort of mental disorder, a lower percentage must be expected to correlate with a lack of criminal capacity at the time of the event. Similarly, a local study in Turkey showed that only 4% of the perpetrators had a lack of criminal capacity [17]. In two different studies, Shaw et al. found that 34% and 44% of those convicted of homicide had recorded mental disorders [32,33]. However, this percentage decreases in the femicide studies. One possible (and limited) explanation of this decrease may be the absence of records regarding alcohol and substance use in any of the files in Shaw et al.’s cases. In our opinion, a variety of predisposing factors and criminal circumstances affect homicide rates and reveal the differences across diverse societies [34]. For example, in a Finnish study, Eronen et al. asserted that mental disorder prevalence among assaulters was high in societies with low homicide rates [35]. In 2011, 14,213 homicide cases were documented in Turkey, corresponding to a 0.19x10^-3^ ratio in the Turkish population, which can be accepted as high. This social tendency towards homicide may help explain some part of this low proportion of perpetrators with mental problems [36,37]. Our results show that the mental disorders of perpetrators may not be a prominent risk factor for femicide in Turkey, but additional studies are needed to confirm this finding.

In our sample, eight perpetrators (10%) were using alcohol and cannabis at the time of the femicide. Similarly, alcohol was detected in eight (13.7%) perpetrators in another study from Turkey [17]. Therefore, drugs clearly did not play an important role in femicide cases in our sample.

In our study, slightly more than 40% of both IPF and non-IPF perpetrators had previous criminal records. This proportion can be accepted as high compared with that in the general population. Since previous arrests/convictions for any type of crime has been found to constitute a risk factor for future offences, including IPV, we have not found any difference between IPF and non-IPF perpetrators [9,12,28]. Criminal records must be a risk factor for femicide cases, but additional studies are needed to elucidate the differences between femicides, ordinary homicides and other crimes.

Previous life threats were more likely in IPF cases than in the non-IPF group, and they were observed in more than 25% of the IPF cases in our study. Death threats were found to be associated with substantially higher risks of femicide [9].

### The victim

Jealousy/infidelity, followed by altercations, were found to be the most common motive for femicide. More common than motives related to separation, jealousy/infidelity was also a leading motive in IPF. These honour killings were not limited to partners, and killing by close relatives/siblings was also found in the non-IPF group. In contrast to our study, several studies in the literature have shown that the most frequently alleged motive for IPF is separation (threats, attempts or consummation), followed by jealousy/infidelity [12]. Moreover, a Turkish study showed that the most common motives for femicide were separation (28.4%) and jealousy/infidelity/honour killings (26.9%); however, this study did not differentiate between IPF/non-IPF; all femicide cases were included in the dataset [15]. A recent study in Turkey showed that envy/honour and custom-related killings accounted for 48% of all femicide cases [17]. Honour killings have historically occurred in many deeply patriarchal cultures, including Turkey [4]. We argue that “so-called honour killings” increased the number of jealousy/infidelity/honour killings; this category was larger than the separation category. Ninety per cent of the victims in our study were killed within four months of their separation, which is a shorter period than reported in some studies [12]. This finding can be interpreted to show that women are at highest risk of femicide in the first four months following their separation.

The “separation” and “jealous/infidelity” motive groups clearly had a lower mean victim age than the other groups. In theft cases, the mean age of the victims increased (approximately 50 years old), whereas the mean age of the perpetrators decreased (28 years old). Theft and domestic altercations were the main motives for the killing of victims older than 50 years of age. In light of these results, if a woman older than 50 years of age is found murdered, the perpetrators can be credibly suspected to be strangers or distant relatives/friends rather than partners or close relatives, and the main motives are likely to be altercations or theft.

A good predictor of female partner homicide is previous violence [38]. Our data showed similar results; some record of previous physical abuse was found in one-fourth of the cases. By contrast, sexual abuse rates seemed very low. There was no intergroup difference in terms of sexual and emotional abuse, which may be linked to little understanding or recognition of in-marriage sexual abuse and the under-reporting of sexual assaults [39]. Of 161 cases, only six involved current sexual abuse; therefore, in femicide cases, sexual abuse do not appear to often be the reason for or method of killing. However, these findings also demonstrated the importance of making it easy for the victim to report such events and of properly evaluating abuse cases. When analysing the low level of previous violence found in our dataset, two points should be considered. First, the information about previous violence in our dataset came from formal sources. Second, only a fraction of gender-based violence is reported to formal sources in Turkey and other developing countries [40,41].

We have limited data about the education level of victims, as court files do not contain this information in most cases. We also found that most women (87.5%) were not employed in a paid job (they were housewives or unemployed), and similar results have been found in several studies conducted in Turkey [14,17], despite the existence of studies contradicting these findings. For example, Karbeyaz et al. claimed that less than the 50% of victims were housewives [15].

Victims are usually mentally competent and are not under the influence of substances or alcohol—at least not at the time of the murder. Alcohol and drug abuse are known risk factors for IPV [42]. However, alcohol was detected in the blood of only two victims. This finding could be related to alcohol consumption in Turkey, which boasts the lowest rate of consumption in Europe, especially among women [43].

### Femicide

Our results showed that the use of all types of firearms (e.g., handguns and shotguns) and edged and pointed weapons was the dominant method of killing in femicides. Similar results have been found in several other studies [12]. Although deaths from the edged and pointed weapons were distributed equally between the IPF and non-IPF groups, handgun use was more common in the former. Indeed, the perpetrator’s legal possession of a firearm is the one of the highlighted risk factors for femicide [9]. Moreover, our results reflect that gaining access to handguns and other firearms is relatively easy in Turkey. Similarly, recent studies from Turkey have shown very similar results; firearm injuries (43–56%) were most common, followed by sharp force injuries (23–28%) [14,17,18].

According to our results, the thorax and head/neck area were the most common wound sites on both IPV and non-IPV victims. There was no difference between IPF and non-IPF injuries in terms of the method, anatomical location and number of injuries. Contradicting the findings of this study, the authors of studies in Taiwan and Portugal reported that the face and/or upper limbs were the most frequently targeted areas in femicide cases [12,28], which may relate to the differences in violent culture/motives between different countries. This assumption is supported by some other findings in the Taiwan study that are not in line with ours, such as the high number of blunt traumatic lesions and the low percentage of firearm deaths. As we will mention below, injury and death rates for blunt trauma have been found to be low in our study and other Turkish studies; in general, killing is the prime intention of femicides in Turkey [18].

Another striking point is that all but one of the perpetrators older than 51 years of age usually used guns and did not commit the murders via blunt trauma, such as beating or strangulation. This finding probably reflects that the physical power and effort required to beat or strangle someone to death are more taxing for older perpetrators. In fact, the 55-year-old perpetrator who strangled two victims only did so after they had been sedated with chloroform. This tendency is also in line with reports about gun use and the ages of perpetrators or victims [44]. Therefore, young perpetrators should probably be investigated first if a woman has been murdered by blunt trauma.

In 28 cases, the victims died due to blunt trauma, but only half of these victims died due to a blunt traumatic lesion. In femicide cases, especially in the IPF context, the perpetrators seemingly act with the intent to kill. This assumption was supported by the high number of wounds in critical regions, such as the head/neck and thorax regions, in assaults committed with firearms and edged and pointed weapons. In addition, the median number of blunt injuries (only 2) and the number of victims with widely distributed blunt traumatic lesions, such as ecchymosis or lacerations, were relatively low for a struggle or simple physical violence. Therefore, femicide cases should be included in a specific “Violence to Women” category. Particularly partners and close relatives (such as siblings) have the intent to kill their victims rather than simply causing them pain. Femicide is usually the ultimate form of punishment in which perpetrators kill without a physical struggle, and typical findings of physical abuse were absent in most cases. Clearly, most of the femicides recorded here were decisive actions: deliberate “executions” rather than manslaughter. These findings are very similar to the assumption made by Goussinsky et al. [45].

Although there is not a precise definition for “overkill” nor an established method for its identification [46], overkill is another characteristic of IPF [47]. Our results showed that more than 1 in 3 IPF victims suffered from overkill. However, in our sample, 28% of non-IPF victims suffered from overkill. These data show that although overkill is more common in IPF cases, it is still prevalent in all kinds of femicide.

### Limitations

The main limitation of this study is its cross-sectional nature; hence, we were only able to report associations rather than definitive temporal or causal relationships. Another important limitation is that the analyses were based on criminal court case files and police records, which may not include health-related issues for both the victims and perpetrators, as well as homicide/suicide cases. The methodology was the strongest feature of the study, as we had an adequate sample size and a proper sampling method and we covered all of Turkey.

### Conclusion

As the WHO noted, the surveillance and screening of femicide and IPV is particularly important in fighting femicide. As the EU-funded project “Femicide across Europe” suggested, a “European Observatory” and other observatories on femicide should be established [2,48]. A recent report underlined that strategic evaluations must be done, especially in two domains: political action and technical steps [49].

Second, training and sensitizing health staff to improve their awareness of the situation is another important step. Most intimate partner homicides are known involve physical abuse, and health staff are the first people to encounter physically abused women [22]. In particular, general practitioners and forensic pathologists may spot at-risk women who show signs of any type of abuse. Moreover, evidence has shown that health staff should be encouraged to participate advocacy interventions [50].

Third, reducing gun ownership is another important step, as mostly firearms are used in IPF cases.

## Supporting information

S1 Table Number of injuires with regard to body regions and assault types. NI: Number of injuries. NW: Number of wounded person. MIC: Mean Injury Count (NI/NW).
(DOCX)
