# Supplementary material for: Femicide in Turkey between 2000 and 2010
Source: PLoS One. 2017 Aug 23;12(8):e0182409. doi: 10.1371/journal.pone.0182409 (PMC5568387; doi:10.1371/journal.pone.0182409)
Supplement: S1 Table — NI: Number of injuries. NW: Number of wounded person. MIC: Mean Injury Count (NI/NW). (DOCX) [file pone.0182409.s001.docx]

S1 table. Number of injuries with regard to body regions and assault types. NI: Number of injuries. NW: Number of wounded person. MIC: Mean injury count (NI/NW)

|  | Handgun | | | Shotgun | | | Knifegun Hachette | | | Blunt  Trauma | | | Ligature Mark | | | TOTAL | | |
| --- | --- | --- | --- | --- | --- | --- | --- | --- | --- | --- | --- | --- | --- | --- | --- | --- | --- | --- |
|  | NI | NW | MIC | NI | NW | MIC | NI | NW | MIC | NI | NW | MIC | NI | NW | MIC | NI | NW | MIC |
| Head  non IPV  IPV |  |  |  |  |  |  |  |  |  |  |  |  |  |  |  |  |  |  |
|  | 14 | 8 | 1.75 | 5 | 5 | 1.00 | 27 | 5 | 5.40 | 44 | 15 | 2.93 | - | - | - | 90 | 33 | 2.72 |
|  | 32 | 26 | 1.23 | 5 | 3 | 1.66 | 53 | 9 | 5.88 | 21 | 11 | 1.90 | - | - | - | 111 | 49 | 2.26 |
| Neck  non IPV  IPV |  |  |  |  |  |  |  |  |  |  |  |  |  |  |  |  |  |  |
|  | 4 | 4 | 1.00 | 5 | 4 | 1.25 | 39 | 13 | 3.00 | 22 | 9 | 2.44 | 6 | 6 | 1.00 | 76 | 36 | 2.11 |
|  | 6 | 4 | 1.50 | 1 | 1 | 1.00 | 45 | 12 | 3.75 | 14 | 5 | 2.80 | 3 | 3 | 1.00 | 69 | 25 | 2.76 |
| Thorax  non IPV  IPV |  |  |  |  |  |  |  |  |  |  |  |  |  |  |  |  |  |  |
|  | 10 | 5 | 2.00 | 18 | 8 | 2.25 | 115 | 23 | 5.00 | 13 | 5 | 2.60 | - | - | - | 156 | 41 | 3.80 |
|  | 27 | 11 | 2.45 | 7 | 5 | 1.40 | 132 | 24 | 5.50 | 7 | 3 | 2.33 | - | - | - | 173 | 43 | 4.02 |
| Abdomen  non IPV  IPV |  |  |  |  |  |  |  |  |  |  |  |  |  |  |  |  |  |  |
|  | 2 | 2 | 1.00 | 5 | 3 | 1.66 | 45 | 12 | 3.75 | 10 | 2 | 5.00 | - | - | - | 62 | 19 | 3.26 |
|  | 8 | 2 | 4.00 | 2 | 2 | 1.00 | 74 | 18 | 4.11 | 1 | 1 | 1.00 | - | - | - | 85 | 23 | 3.69 |
| Pelvis  non IPV  IPV |  |  |  |  |  |  |  |  |  |  |  |  |  |  |  |  |  |  |
|  | - | - | - | 1 | 1 | 1.00 | 1 | 1 | 1.00 | 3 | 2 | 1.50 | - | - | - | 5 | 4 | 1.25 |
|  | - | - | - | - | - | - | 3 | 2 | 1.50 | - | - | - | - | - | - | 3 | 2 | 1.50 |
| Upper Extremities  non IPV  IPV |  |  |  |  |  |  |  |  |  |  |  |  |  |  |  |  |  |  |
|  | 1 | 1 | 1.00 | 6 | 5 | 1.12 | 33 | 12 | 2.75 | 27 | 6 | 4.50 | 1 | 1 | 1.00 | 68 | 25 | 2.72 |
|  | 13 | 9 | 1.44 | 3 | 3 | 1.00 | 66 | 13 | 5.07 | (4 | 2 | 2.00 | 1 | 1 | 1.00 | 87 | 28 | 3.10 |
| Lower Extremities  non IPV  IPV |  |  |  |  |  |  |  |  |  |  |  |  |  |  |  |  |  |  |
|  | 2 | 1 | 2.00 | 2 | 2 | 1.00 | 29 | 8 | 3.62 | 25 | 9 | 2.77 | 1 | 1 | 1.00 | 59 | 21 | 2.80 |
|  | - | - | - | 1 | 1 | 1.00 | 46 | 11 | 4.18 | 4 | 2 | 2.00 | 1 | 1 | 1.00 | 52 | 15 | 3.46 |
| Total |  |  |  |  |  |  |  |  |  |  |  |  |  |  |  |  |  |  |
| non IPV | 33 | 21 | 1.57 | 42 | 28 | 1.50 | 289 | 74 | 3.90 | 144 | 48 | 3.00 | 8 | 8 | 1.00 | 516 | 179 | 2.88 |
| IPV | 86 | 52 | 1.65 | 19 | 15 | 1.26 | 419 | 89 | 4.70 | 51 | 24 | 2.12 | 5 | 5 | 1.00 | 580 | 185 | 3.13 |
